# Tissue proteome analysis for profiling proteins associated with lymph node metastasis in gallbladder cancer

**DOI:** 10.1186/s12885-023-10840-3

**Published:** 2023-05-04

**Authors:** Vaishali Jain, Javed Akhtar, Ratna Priya, Puja Sakhuja, Surbhi Goyal, Anil Kumar Agarwal, Vivek Ghose, Ravindra Varma Polisetty, Ravi Sirdeshmukh, Fouzia Siraj, Poonam Gautam

**Affiliations:** 1 ICMR-National Institute of Pathology, Safdarjung Hospital Campus, New Delhi, 110029 India; 2 Manipal Academy of Higher Education (MAHE), Manipal, 576104 India; 3 Jamia Hamdard-Institute of Molecular Medicine, Jamia Hamdard, New Delhi, 110062 India; 4 Govind Ballabh Pant Institute of Postgraduate Medical Education and Research (GIPMER), New Delhi, 110002 India; 5 Institute of Bioinformatics, International Tech Park, Bangalore, 560066 India; 6 Department of Biochemistry, Sri Venkateswara College, University of Delhi, New Delhi, 110021 India

**Keywords:** Gallbladder carcinoma, Lymph node metastasis, Tissue proteomics, iTRAQ

## Abstract

Lymph node (LN) metastasis is the earliest sign of metastatic spread and an established predictor of poor outcome in gallbladder cancer (GBC). Patients with LN positive GBC have a significantly worse survival (median survival- 7 months) than patients with LN negative disease (median survival- ~ 23 months) in spite of standard treatment which includes extended surgery followed by chemotherapy, radiotherapy and targeted therapy. This study aims at understanding the underlying molecular processes associated with LN metastasis in GBC. Here, we used iTRAQ-based quantitative proteomic analysis using tissue cohort comprising of primary tumor of LN negative GBC (*n* = 3), LN positive GBC (*n* = 4) and non-tumor controls (Gallstone disease, *n* = 4), to identify proteins associated with LN metastasis. A total of 58 differentially expressed proteins (DEPs) were found to be specifically associated with LN positive GBC based on the criteria of *p* value ≤ 0.05, fold change ≥ 2 and unique peptides ≥ 2. These include the cytoskeleton and associated proteins such as keratin, type II cytoskeletal 7 (KRT7), keratin type I cytoskeletal 19 (KRT19), vimentin (VIM), sorcin (SRI) and nuclear proteins such as nucleophosmin Isoform 1 (NPM1), heterogeneous nuclear ribonucleoproteins A2/B1 isoform X1 (HNRNPA2B1). Some of them are reported to be involved in promoting cell invasion and metastasis. Bioinformatic analysis of the deregulated proteins in LN positive GBC using STRING database identified ‘neutrophil degranulation’ and ‘HIF1 activation’ to be among the top deregulated pathways. Western blot and IHC analysis showed a significant overexpression of KRT7 and SRI in LN positive GBC in comparison to LN negative GBC. KRT7, SRI and other proteins may be further explored for their diagnostics and therapeutic applications in LN positive GBC.

**Supplementary Information:**

The online version contains supplementary material available at 10.1186/s12885-023-10840-3.

## Introduction

Lymph node (LN) metastasis is the initial sign of metastasis and is one of the established predictors of prognosis in gallbladder cancer (GBC) patients. As per AJCC, 8^th^ Edition [1], GBC stage III is catergorized into Stage IIIA (LN negative) and IIIB (LN positive) based on LN status. In LN negative GBC, tumor is spread to liver and/or one nearby extrahepatic organ but not to nearby lymph nodes, while in LN positive GBC, tumor cells disseminates to nearby lymph nodes. After the spread of tumor cells to lymph nodes, the survival of the patients is significantly worse [2]. Five-year survival rates of GBC cases with node negative pN0 was reported to be ~ 80%, while for node positive pN1 and pN2 was ~ 57% and ~ 23% respectively [3, 4]. Negi et al. reported that lymph node ratio (LNR- ratio of metastatic and total number of examined nodes) independently may predict survival after resection [5]. Tumor recurrence is common in LN positive GBC inspite of standard treatment as there is no effective targeted therapy available for these patients. Molecular insights into the mechanisms associated with survival and growth of tumor cells in LNs may be useful for improved therapy to combat LN metastases.

Recent findings provide understanding of the mechanisms causing growth of tumor cells in LNs. These tumor cells as well as the molecules secreted by them decrease anti-tumor immunity and promote tumor growth via engagement of stromal, lymphoid and myeloid cells present in the organ-restricted tumor as well as in the lymphatic system. Vascular endothelial growth factors (VEGFs) e.g. VEGF-C, VEGF-D and several chemokines such as CCL21, CXCL12 and CCL1 facilitate LN metastasis in several cancers [6]. The levels of tissue and serum VEGF-C and D are increased in GBC patients and promote LN metastasis [7-9]. Yao et al.detected the expression of nuclear chemokine receptor type 4 (CXCR4) in the nucleus and/or cytoplasm of GB cancer cells and is reported to be associated with LN metastases [10]. CCR7 is found to be overexpressed in GBC and modulates TNF-α-induced LN metastasis of GBC [11]. Shah et al.evaluated cytokeratin CK7 as marker for detecting micrometastatic disease in lymph nodes (LN with 0.2–2 mm tumor size) using IHC analysis and found low incidence rates (7.5%) of LN micrometastasis in GBC [12].

Application of high throughput proteomic approaches have been successful to understand the complex biological processes and has been applied to understand the molecular processes involved in LN metastasis in various cancers [13-15]. However, there is no high throughput proteomic analysis carried out to study LN metastasis in GBC till date. The present study applied iTRAQ-based quantitative proteomics to identify differentially expressed proteins (DEPs) in primary tumor of LN positive GBC in comparison to LN negative GBC and gallstone disease (GSD) as non-tumor controls. Further, bioinformatic analysis was performed to identify deregulated processes, pathways and networks. The expression of some of the functionally relevant proteins was verified by Western blot analysis.

## Methodology

### Clinical samples

Adult patients with age ≥ 20 years diagnosed with GBC or GSD cases (non-tumor control) visiting Govind Ballabh Pant Institute of Postgraduate Medical Education and Research (GIPMER), New Delhi, were recruited for the study after approval from the Institutional Human Ethics Committee [Maulana Azad Medical College- Institutional Ethics Committee and ICMR-National Institute of Pathology- Institutional Ethics Committee, New Delhi]. Inclusion criteria includes (1) Patients ≥ 20 year age (2) GBC cases with adenocarcinomas. Exclusion criteria includes (1) Patients < 20 year age (2) GBC cases other than adenocarcinomas (3) presence of biliary tract or any other malignancy other than gallbladder carcinoma (4) Patients who are morbidly ill or having other malignancies (5) Those who have already taken the treatment. Tumor Staging was done on the basis of clinical data of patients, histopathological evaluation and imaging tools, as per AJCC, 8^th^ edition staging system [1]. The tissue sample from GBC cases had ~ 70% tumor cells. Fresh frozen tissue samples from LN negative GBC cases (*n* = 4), LN positive GBC cases (*n* = 4) and GSD cases with no dysplasia as ‘non-tumor controls’ (*n* = 5) were used in this study. Tissue samples were collected immediately after surgical resection from patients with GBC or GSD and stored at -80° C until used for further analysis. Formalin-fixed tissue samples from LN negative GBC cases (*n* = 15), LN positive GBC cases (*n* = 15) and GSD cases with no dysplasia as ‘non-tumor controls’ (*n* = 15) were used for immunohistochemistry (IHC) analysis. Clinico-pathological data of these subjects are detailed in Table 1. Clinical parameters such as TNM, Stage, grade, white cell count, liver enzymes (AST/ ALT/ ALP), bilirubin and co-morbidities (jaundice, diabetes melitus, hypertension, loss of appetite and loss of weight) for the GBC patients and control groups as available (~ 68%) are provided in Supplementary Table S1.

**Table 1:** Clinico-pathological parameters of the patients used for the study

	Total number	Number of males	Number of females	Mean age (Years)	Age range (years)
Total GBC Cases	30	3	27	54.5	32–86
Stages					
GBC, Stage II	5	0	5	56.6	35–86
GBC, Stage IIIA	10	1	9	51.3	40–74
GBC, Stage IIIB	12	1	11	54.0	40–72
GBC, Stage IV	3	1	2	55	38–66
Histological grade					
Well-differentiated (G1)	1	0	1	–-	–-
Moderately-differentiated (G2)	26	2	24	–-	–-
Poorly-differentiated (G3)	3	1	2	–-	–-
LN status					
LN Negative	15	1	14	–-	–-
LN Positive	15	2	13	–-	–-
Controls (GSD)	15	2	13	40.8	22–56

### Protein extraction

Tissue from individual cases [tumor tissue from GBC patients] or controls [GB tissue from GSD cases] was grinded in liquid nitrogen followed by addition of modified RIPA buffer with 2% protease inhibitor cocktail. The tissue homogenate was then sonicated and centrifuged at 13,000 g for 20 min at 4 °C. The supernatant was collected and protein estimation was done using Bradford assay. SDS-PAGE was performed to analyze the protein profile of the tissue lysate from different groups and normalized the protein concentration based on total density [16].

### iTRAQ Labeling

For iTRAQ experiments, a pool of GSD tissue lysate (*n* = 4) was used as control while individual tissue lysate from GBC cases (*n* = 3 for stage IIIA and *n* = 4 for stage IIIB) was used for the quantitative proteomic analysis. The experimental design is shown in Supplementary Fig. S1.

In brief, proteins (100 µg) from control (*n* = 4, pooled sample) and LN Negative GBC (Stage IIIA, *n* = 3, individual samples) and LN Positive GBC (Stage IIIB, *n* = 4, individual samples) was reduced, alkylated and digested with trypsin followed by labelling of peptides with 8-plex iTRAQ reagents separately with specific iTRAQ labels (Reagent 113, 114, 115, 116, 117, 118, 119 and 121) as per the manufacturer’s instructions (iTRAQ Reagents Multiplex kit; Applied Biosystems) [16]. The labeled samples were pooled vacuum-dried and subjected to strong cation exchange (SCX) clean up (Cation exchange cartridge, Sciex, US), and desalted using C18 column (Zorbax 300SB-C18, Agilent Technologies, US) as per the manufacturer’s instructions. The samples were then vacuum-dried and used for mass spectrometric analysis (nano-LC MS/MS analysis) [16].

### LC–MS/MS analysis

Nanoflow electrospray ionization tandem mass spectrometric analysis was carried out using Orbitrap Fusion (Thermo Scientific, Bremen, Germany) interfaced with Easy-nLC 1000 nanoflow LC system [16]. Peptides from each sample were enriched using a C18 trap column (75 μm × 2 cm) at a flow rate of 3 μl/min and fractionated on an analytical column (75 μm × 50 cm) at a flow rate of 280 nl/min using a linear gradient of 8–60% acetonitrile (ACN) over 46 min. Mass spectrometric analysis was performed in a data dependent manner with a cycle time of 3 s using the Orbitrap mass analyzer at a mass resolution of 120,000 at m/z 200. For each MS cycle, top most intense precursor ions were selected and subjected to MS/MS fragmentation and detected at a mass resolution of 50,000 at m/z 200. The fragmentation was carried out using higher-energy collision dissociation (HCD) mode. Normalized collision energy (CE) of 30% was used to obtain release of reporter ions from all peptides detected in the full scan. The ions selected for fragmentation were excluded for next 30 s. The automatic gain control for full FT MS and FT MS/MS was set to 3e^6^ ions and 1e^5^ ions respectively with a maximum time of accumulation of 50 ms for MS and 75 ms for MS/MS. The lock mass with 10 ppm error window option was enabled for accurate mass measurements [16]. The LC–MS/MS analysis was performed in four replicates.

### Identification and quantification of proteins

Protein identification, quantification and annotations of DEPs were carried out as described earlier by Priya et al. [17]. The MS/MS data was analyzed using Proteome Discoverer (Thermo Fisher Scientific, version 2.2) with Mascot and Sequest HT search engine nodes using NCBI RefSeq database (release 89). Search parameters included trypsin as the enzyme with 2 missed cleavage allowed; precursor and fragment mass tolerance were set to 10 ppm and 0.1 Da, respectively; Methionine oxidation and deamidation of asparagines and glutamine amino acids was set as a dynamic modification while methylation modification at cysteine and iTRAQ modification at N-terminus of the peptide and lysines were set as static modifications. The peptide and protein information were extracted using high peptide confidence and top one peptide rank filters. The FDR was calculated using percolator node in proteome discoverer 2.2. High confidence peptide identifications were obtained by setting a target FDR threshold of 1% at the peptide level.

The iTRAQ intensity of proteins from each of the four replicates was used for the PCA plot analysis using MetaboAnalyst 5.0 [18] to determine the correlation among the four replicates dataset as well as the correlation of GSD, LN Negative and LN Positive proteome dataset.

Relative quantitation of proteins was carried out based on the intensities of reporter ions released during MS/MS fragmentation of peptides. The proteins identified in all the four replicates were used for the analysis. The average relative intensities of the two reporter ions for each of the unique peptide identifiers for a protein were used to determine relative quantity of a protein and percentage variability. Proteins identified with ≥ 2 unique peptides, with two-fold change or above and FDR adjusted *p* value < 0.05 were considered significant and used for further analysis [16]. The volcano maps for ‘LN negative GBC vs GSD’ and ‘LN positive GBC vs GSD’ were prepared by using log2 fold change and -log10 (*p*-value) as the co-ordinates and significant fold change ≥ 2.0 and *p*-value < 0.05 were considered to screen the proteins.

The data was analyzed for DEPs in individual patient with LN metastasis or without LN metastasis vs GSD (non-tumor control) and represented as Venn diagram. Further, the list of DEPs in LN positive GBC was derived and used for bioinformatics analysis.

### Bioinformatic analysis

STRING (http://www.string-db.org) is a database to visualise protein–protein interaction including physical and functional interactions. Mapping of DEPs in LN positive GBC for localization, associated molecular functions, pathways and protein–protein interaction analysis was performed using the STRING (Search Tool for the Retrieval of Interacting Genes/Proteins) database by setting up the parameters as *Homo Sapiens* and combined confidence score greater than 0.4 [19].

### Western blot analysis

Proteins were separated by SDS-PAGE and electrotransferred to polyvinyl difluoride (PVDF) membrane [20]. The blots were blocked with 5% skimmed milk powder in TBST [1 × tris buffered saline (10 mM Tris–Cl, pH 7.4 and 30 mM NaCl) with 0.05% Tween 20 and 0.005% Triton-X-100] at RT for 1 h. The blots were then incubated with primary antibodies against Keratin, type II cytoskeletal 7 (KRT7) (1:1000, Cat. No. MA5-11,986, Thermo Scientific, USA) and keratin type I cytoskeletal 19 (KRT19) (1:500, Cat No. MA5-12,663, Thermo Scientific, USA), Sorcin (SRI) (1:1000, Cat. no. PA5-23,143, Thermo Scientific, USA) and Nucleophosmin 1 (NPM1) (1:1000, Cat No. MA5-17,141, Thermo Scientific, USA) at 4 °C overnight. Secondary antibodies against rabbit IgG (1:20,000, Thermo Scientific, USA) was used for SRI while mouse IgG (1:20,000, Thermo Scientific, USA) was used for KRT7, KRT19 and NPM1 respectively at RT for 1 h. The blots were developed using the enhanced chemiluminescent (ECL) Kit (Millipore, USA). The images were acquired using Chemidoc MP imager and immunoblots were analyzed using Image Lab 4.1 software (Bio-Rad, USA). Densitometric analysis of the specific band showing reactivity was performed to get relative expression of target proteins in LN positive (*n* = 4) vs LN negative (*n* = 4) and LN positive (*n* = 4) vs GSD (non-tumor control) (*n* = 4). The maximum density of GSD cases was used to calculate the fold change in expression in LN positive GBC. Statistical analysis was performed using GraphPad Prism 5 [21]. Differences in expression of target proteins between LN positive vs LN negative, LN positive GBC vs GSD and LN negative GBC vs GSD were tested with unpaired t-test (two-tailed) with confidence intervals of 95%. The *p*-values less than 0.05 was used to indicate statistical significance.

### Immunohistochemistry analysis

IHC was performed on FFPE tissues using individual tissue sections from non-tumor controls, GSD cases (*n* = 15), LN negative GBC (*n* = 15) and LN positive GBC cases (*n* = 15) (Supplementary Table S1) to analyze the expression of KRT7, KRT19 and SRI protein. IHC analysis was performed as described earlier by Akhtar et al*.* [20]. In brief, after deparaffinization and rehydration of FFPE tissue sections, antigen retrieval was performed by immersing the slide in antigen retrieval buffer (20 mM Tris buffer, pH 9.0) at 90 °C for 20 min. Endogenous peroxidases were blocked with 0.03% hydrogen peroxide, and nonspecific binding was blocked with protein blocking reagent. Sections were then incubated for 1 h at RT with primary antibody against KRT7 (dilution 1:250, catalogue no. 307 M-96, Merck, USA), KRT19 (ready-to-use, catalogue no. PR138, Pathnsitu Biotechnologies, USA) and SRI (dilution 1:3000, catalogue no. PA5-23,143, Thermo Scientific, USA) followed by incubation with PolyExcel PolyHRP for 40 min at RT. Tissue sections were then incubated with Stunn DAB working solution for 5 min at RT (PathnSitu Biotechnologies, USA). Sections were counter stained with Mayer’s hematoxylin, dehydrated and images were taken under the microscope. The distribution of staining and staining intensity across the section was observed under the microscope. The scoring criteria were based on both staining intensity and distribution. For KRT7, KRT19 and SRI, the 2 + or higher intensity, with ≥ 25% distribution was considered as ‘Positive’, while 1 + positivity or < 25% distribution was considered as ‘Negative’. The data was analyzed for nuclear expression only as well as both nuclear and cytoplasmic expression for SRI. IHC data analysis was done by two independent pathologists. The statistical analysis (Chi square test, two-tailed) was performed using GraphPad Prism 5 [21] to study the correlation of KRT7, KRT17 and SRI expression among LN positive vs LN negative GBC, LN positive vs GSD and LN negative vs GSD. The *p*-value less than 0.05 indicated statistical significance.

## Results

**Fig. 1:**
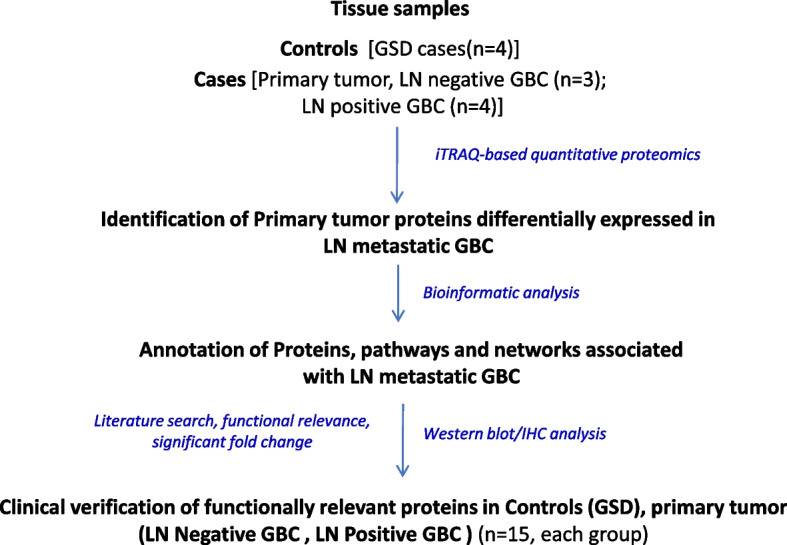
Overall workflow of the study. GSD- Gallstone disease; GBC- Gallbladder carcinoma, LN- Lymph node

In the present study, we performed the differential tissue proteome profiling to identify the proteins and associated molecular processes and pathways in LN positive GBC followed by verification of selected proteins based on their significant fold change in LN metastatic GBC and their functional association with LN metastasis using Western blot and/or IHC analysis. The overall work plan of the study is shown in Fig. 1.

### Identification of differentially expressed proteins in LN positive GBC

Tissue lysate was prepared using individual samples followed by protein estimation by Bradford assay. SDS-PAGE analysis was performed to ensure equal loading using densitometric analysis (Supplementary Fig. S2). Further, iTRAQ based quantitative proteomic analysis to identify proteins associated with LN metastasis in GBC.

**Fig. 2:**
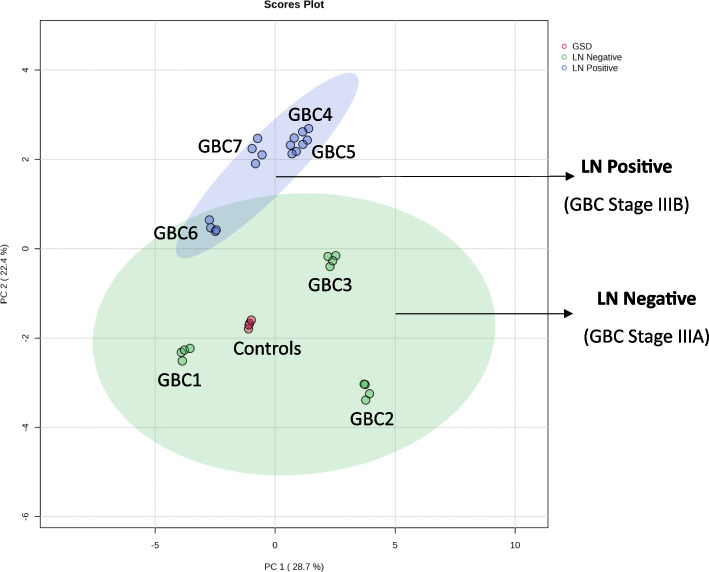
Principal component analysis for the control, LN negative and LN positive cases. We observed a significant correlation among the four technical replicates for each sample and found the majority of the LN negative cases group together. PCA was performed based on the log2-transformed intensity of proteins identified in the four replicates. Green area is depicting the LN negative and blue area indicates LN positive GBC. Red dots indicate controls. The PCA plot is derived using the iTRAQ reporter intensity from the quantitative proteomics data using metaboanalyst

The mass spectrometric analysis led to the identification of a total of 1076 proteins. A comparison of four technical replicates of mass spectrometric runs was performed and the data is presented as Venn diagram (Supplementary Fig. S3). The analysis revealed around 700 proteins in each replicate run and a total of 468 proteins were found to be common among the four replicates. PCA plot analysis for the assessment of replicate data showed a significant correlation among the four replicates for each sample (Fig. 2). We observed the non-tumor control group and LN negative GBC to be more similar to each other, while patients with LN positive GBC clustered as a distinct group. We found 129 DEPs in LN Negative GBC and a total of 132 DEPs in LN Positive GBC with ≥ two-fold change and adjusted *p*-value ≤ 0.05.

**Fig. 3:**
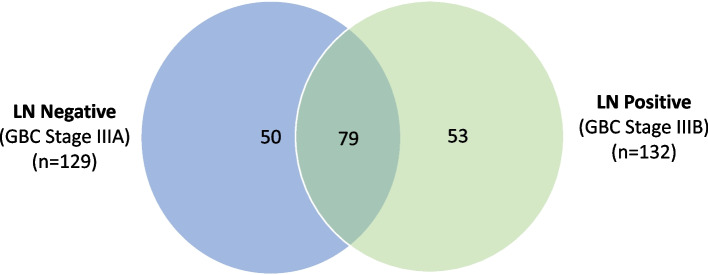
Venn Diagram showing the comparison of DEPs in LN negative and LN positive GBC. A total of 79 proteins are common to both stage LN negative and LN positive GBC, while 50 proteins were specific to LN negative GBC and 53 proteins are specific to LN positive. A total of 5 proteins showed opposite correlation in expression among the proteins common to both the stages. n signifies the number of non redundant proteins in both the stages

The comparison of LN positive and LN negative group showed 53 proteins specific to LN positive GBC while 79 proteins were common between the two stages (Fig. 3). Among the 79 common proteins, expression of 5 proteins showed opposite correlation. Overall, we found 58 proteins (53 + 5) to be differentially expressed in LN positive GBC and the list is provided in Table 2. This includes functionally relevant proteins associated with LN metastasis in cancer such as KRT7, KRT19, SRI, NPM1, Annexin A2 (ANXA2), Annexin A5 (ANXA5). The detailed protein and peptide list is provided in Supplementary Table S2.

**Table 2:** List of 58 proteins differentially expressed in LN positive GBC. This includes 53 proteins specific to LN positive GBC (A) and 5 proteins showed opposite correlation in expression among the proteins common to both the stages (B). The detailed protein and peptide list is provided in Supplementary Table S1

		**LN Negative GBC**			**LN Positive GBC**			
**Gene Symbol**	**Protein Name**	**GBC1**	**GBC2**	**GBC3**	**GBC4**	**GBC5**	**GBC6**	**GBC7**
**(A)**								
A1BG	Alpha-1B-glycoprotein precursor	1.296	0.538	0.51	**0.447**	0.581	0.883	0.734
ADH1C	Alcohol dehydrogenase 1C	0.809	4.096	1.362	0.486	0.656	0.627	**0.448**
AGR2	Anterior gradient protein 2	0.99	0.53	**1.998**	1.582	**4.208**	1.685	0.712
ALDH18A1	Delta-1-pyrroline-5-carboxylate synthase isoform X1	1.126	0.867	1.471	**3.227**	1.416	0.927	1.246
ANP32A	Acidic leucine-rich nuclear phosphoprotein 32 family member A	0.845	0.808	1.534	**2.300**	1.439	1.214	1.219
ANXA10	Annexin A10 isoform X1	0.886	1.017	1.623	**4.130**	1.087	1.283	0.889
AOC3	Membrane primary amine oxidase isoform X1	0.559	0.749	0.856	**0.50**	0.733	0.703	**0.485**
APEX1	DNA-(apurinic or apyrimidinic site) lyase	1.372	0.688	1.223	**2.057**	1.462	1.311	1.311
APOC3	Apolipoprotein C-III precursor	0.68	1.408	0.466	**0.165**	**0.158**	**0.109**	**0.245**
CAPG	Macrophage-capping protein isoform X1	1.274	0.606	0.877	1.363	1.103	1.402	**2.061**
CKMT1A	Creatine kinase U-type, mitochondrial isoform 1 precursor	1.163	0.786	1.367	**3.211**	1.695	1.445	1.014
COL1A2	Collagen alpha-2(I) chain precurso	0.677	0.654	0.623	0.520	**0.486**	0.563	0.621
COX4I1	Cytochrome c oxidase subunit 4 isoform 1, mitochondrial isoform X1	0.712	1.593	1.013	**2.687**	1.265	0.843	1.012
COX5A	Cytochrome c oxidase subunit 5A, mitochondrial precursor	0.652	1.782	1.321	**2.817**	1.397	0.894	1.006
COX6B1	Cytochrome c oxidase subunit 6B1	0.891	1.431	1.406	**2.289**	1.047	1.140	0.977
DLST	Dihydrolipoyllysine-residue succinyltransferase component of 2-oxoglutarate dehydrogenase complex, mitochondrial isoform 1 precursor	0.756	1.553	1.66	**3.701**	**2.754**	0.988	1.215
ECH1	Delta(3,5)-Delta(2,4)-dienoyl-coa isomerase, mitochondrial isoform X1	0.934	1.796	1.434	1.135	**2.459**	0.993	0.957
EEF1A1	Elongation factor 1-alpha 1	0.665	1.741	1.307	**2.511**	1.817	0.666	**3.143**
FABP5	Fatty acid-binding protein 5	0.895	0.545	1.464	0.827	1.438	1.035	**8.795**
GMDS	GDP-mannose 4,6 dehydratase isoform 1	1.033	0.937	1.398	**3.061**	0.816	0.937	0.853
HADHB	Trifunctional enzyme subunit beta, mitochondrial isoform X1	0.988	1.565	1.457	**2.118**	1.514	0.995	1.093
HNRNPA1	Heterogeneous nuclear ribonucleoprotein A1 isoform X1	0.618	0.813	1.613	**3.609**	**3.282**	1.135	**2.160**
HNRNPA2B1	Heterogeneous nuclear ribonucleoproteins A2/B1 isoform X1	0.523	0.831	1.256	**2.594**	**2.939**	0.807	**2.714**
HNRNPA3	Heterogeneous nuclear ribonucleoprotein A3 isoform a	0.699	0.659	1.356	1.376	**3.393**	0.672	**2.360**
HNRNPK	Heterogeneous nuclear ribonucleoprotein K isoform X1	0.91	0.816	1.922	**2.541**	**2.434**	1.183	**2.334**
HSP90AA1	Heat shock protein HSP 90-alpha isoform 1	0.743	0.976	1.611	1.744	**2.088**	1.139	1.574
HSP90AB1	Heat shock protein HSP 90-beta isoform X1	0.802	0.858	1.903	**2.421**	1.800	1.345	**2.728**
HSPA8	Heat shock cognate 71 kda protein isoform X1	0.712	1.162	1.067	1.588	1.588	1.726	**2.270**
HSPA9	Stress-70 protein, mitochondrial precursor	0.796	1.664	1.656	**3.183**	**2.044**	1.037	**2.357**
IDH2	Isocitrate dehydrogenase [NADP], mitochondrial isoform 1 precursor	1.011	1.722	1.231	**2.148**	1.310	0.744	1.181
KRT1	Keratin, type II cytoskeletal 1	1.418	0.585	0.606	**0.473**	0.768	0.611	0.897
KRT17	Keratin, type I cytoskeletal 17	1.088	0.765	1.091	0.827	1.556	**4.911**	**7.180**
KRT19	Keratin, type I cytoskeletal 19	1.002	0.749	1.788	**7.241**	**3.853**	**2.390**	0.930
KRT6A	Keratin, type II cytoskeletal 6A	1.252	0.732	1.397	1.202	0.888	1.352	**2.212**
KRT7	Keratin, type II cytoskeletal 7	0.878	0.72	0.963	**2.651**	1.944	**2.497**	0.820
LASP1	LIM and SH3 domain protein 1 isoform a	1.531	1.045	1.107	**2.971**	1.467	0.881	1.703
LRPPRC	Leucine-rich PPR motif-containing protein, mitochondrial precursor	1.312	0.659	1.544	**3.315**	**2.019**	0.905	1.217
MDH2	Malate dehydrogenase, mitochondrial isoform 1 precursor	0.797	1.519	1.651	**3.556**	**2.404**	1.465	1.694
NPM1	Nucleophosmin isoform 1	0.72	1.224	1.131	**3.362**	0.835	1.725	**2.930**
PGD	6-phosphogluconate dehydrogenase, decarboxylating isoform 1	1.811	0.967	1.04	1.270	1.084	1.132	**2.052**
PKM	Pyruvate kinase PKM isoform c	0.9	0.599	1.345	**2.065**	1.333	1.416	1.940
PPIA	Peptidyl-prolyl cis–trans isomerase A isoform 1	0.733	0.908	1.3	1.778	1.747	1.446	**2.435**
PPIB	Peptidyl-prolyl cis–trans isomerase B precursor	0.746	1.261	1.31	1.577	1.066	1.279	**2.299**
RPS28	40S ribosomal protein S28	0.82	1.712	1.642	**2.046**	1.871	0.681	**2.114**
SERPINH1	Serpin H1 isoform X1	0.988	0.881	1.269	1.046	1.450	1.061	**2.587**
SLC25A3	Phosphate carrier protein, mitochondrial isoform a precursor	0.975	1.608	2.098	**2.371**	1.585	0.895	1.314
SLC25A6	ADP/ATP translocase 3	1.247	1.422	1.776	**2.021**	1.701	0.950	1.430
SRI	Sorcin isoform A	0.868	0.753	1.011	0.828	**2.380**	**2.394**	0.941
TAGLN2	Transgelin-2 isoform	0.566	0.535	0.634	0.965	1.624	1.629	**2.082**
TUFM	Elongation factor Tu, mitochondrial isoform 1 precursor	0.975	0.828	1.582	**3.457**	1.009	0.803	1.609
TYMP	Thymidine phosphorylase isoform 1 precursor	1.97	1.138	1.024	0.905	1.045	1.252	**2.441**
VCAN	Versican core protein isoform 1 precursor	0.626	0.725	0.895	0.690	1.059	**3.567**	0.807
VIM	Vimentin	0.686	0.522	0.685	**0.387**	0.863	1.376	1.479
**(B)**								
ADH4	Alcohol dehydrogenase 4 isoform 1	**0.50**	**9.87**	**2.15**	**0.49**	**0.50**	**0.47**	**0.43**
ANXA2	Annexin A2 isoform 1	0.54	**0.47**	0.58	1.51	1.29	**2.94**	**2.09**
ANXA5	Annexin A5	0.76	**0.40**	0.52	1.60	1.17	**2.75**	1.15
RDH16	Retinol dehydrogenase 16 isoform 1	0.53	3.57	3.54	0.68	**0.49**	**0.39**	**0.50**
UGT2B7	UDP-glucuronosyltransferase 2B7 isoform 1 precursor	0.49	**3.62**	1.34	0.68	**0.49**	0.38	**0.37**

The proteins marked in bold are DEPs with two-fold change, adjusted *p* value < 0.05 and identified with ≥ 2 unique peptides. DEPs- Differentially expressed proteins

In order to screen the most relevant proteins, we applied a criteria of significant fold change (≥ two-fold, *p* value < 0.05) in ≥ 50% LN positive GBC cases and no change in expression level (≤ 1.3 fold change) in LN negative GBC cases and found five proteins (KRT7, KRT17, SRI, NPM1 and HNRNPA2B1) showed overexpression (≥ two-fold, *p* value < 0.05) in ≥ 50% of the LN positive patients and ≤ 1.3 fold change in LN negative patients.

### Bioinformatic analysis

**Fig. 4:**
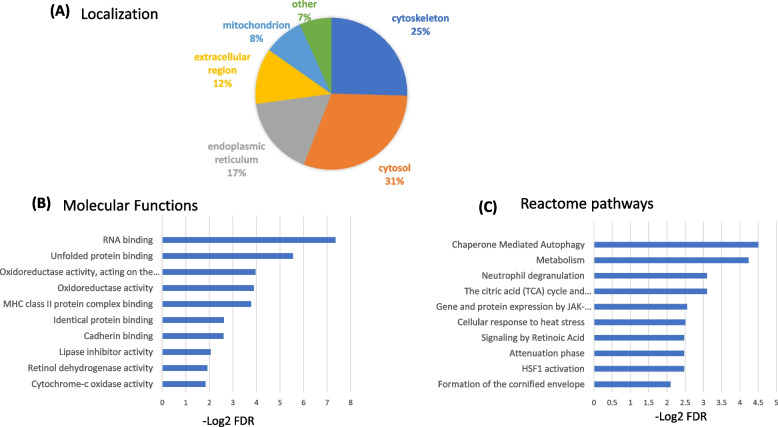
Gene ontology of 58 proteins DEPs in LN positive GBC. (**A**) Subcellular localisation according to UniProt database. (**B**) Molecular functions and (**C**) Reactome pathways according to STRING database

A gene ontology analysis for the localization of 58 DEPs showed that 31% of them belong to cytosol, 25% belong to cytoskeleton, 17% are associated to endoplasmic reticulum, 12% are from extracellular region, 7% are associated to mitochondria and 5% are associated with other localization (Fig. 4A). The top molecular functions include RNA binding, unfolded protein binding, oxidoreductase activity and MHC class II protein complex binding (Fig. 4B, Supplementary Table S3A) and the significantly altered pathways include ‘neutrophil degranulation’ and ‘HIF1 activation’ (Fig. 4C, Supplementary Table S3B). ‘Tumor associated neutrophils’ has been shown to be positively associated with LN metastasis in early gastric cancer [22] ‘neutrophil degranulation’ is reported to be a contributing factor to LN metastasis in breast cancer [23]. Pezutto et al. reported the role of HIF1 activation in cancer progression via regulation of VEGFs and endothelial mesenchymal transition (EMT) related genes [24].

**Fig. 5:**
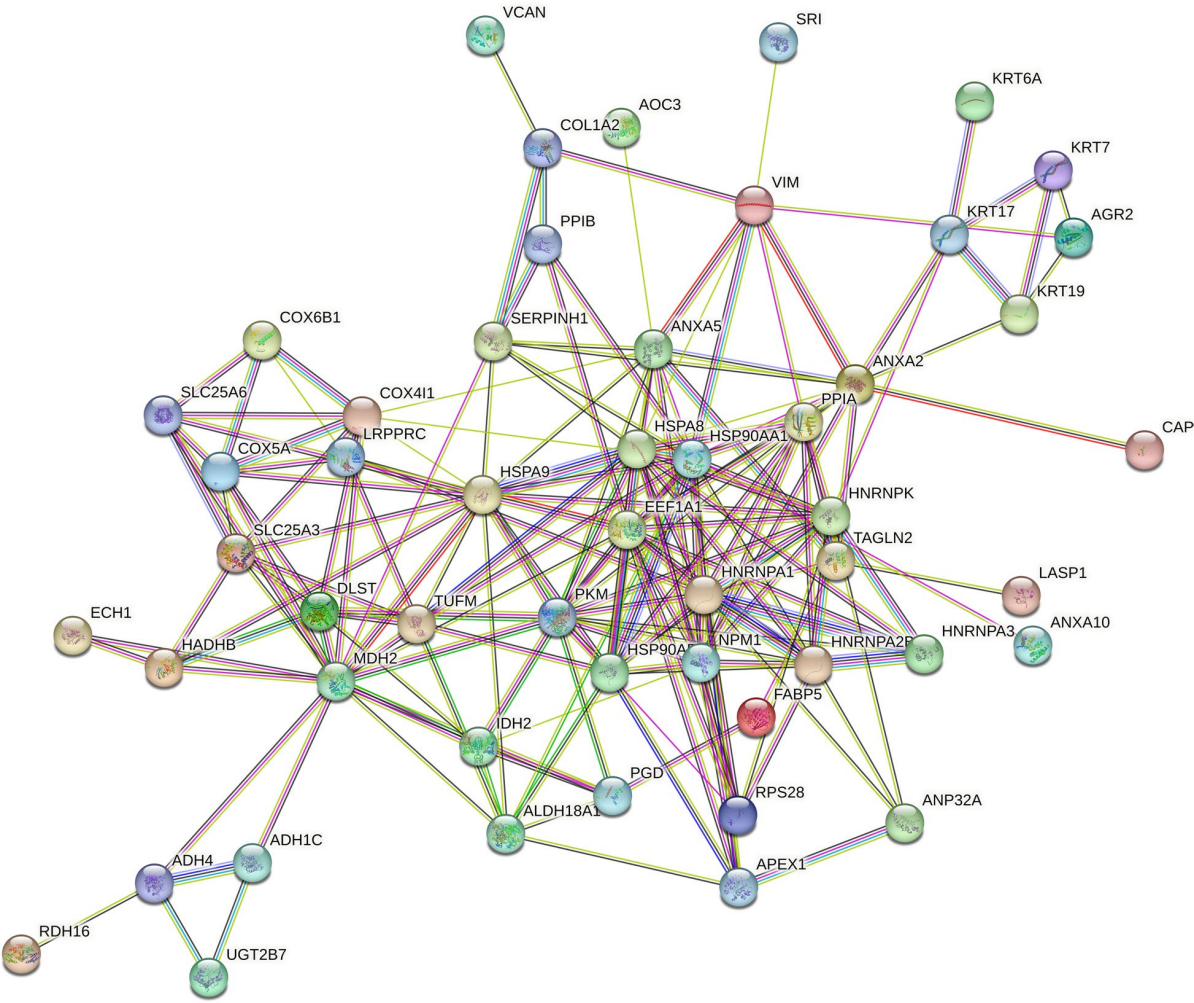
Protein–protein interaction analysis of 58 DEPs in LN positive GBC using STRING database. We observed HSP90AA1, HSPA8, HSPA9, MDH2, PKM, HNRNPA1, HNRNPK, NPM1 and ANXA2 as the hub molecules. The proteins HSPA8, HSPA9, PKM, HNRNPA1, NPM1, ANXA2 are already reported to be associated with LN metastasis in GBC or other cancers

We further analyzed the DEPs within individual patients and the data is represented as Volcano plots in Supplementary Fig. S4. Protein–Protein Interaction Networks using 58 DEPs showed hub molecules including HSP90AA1, HSPA8, HSPA9, MDH2, PKM, HNRNPA1, HNRNPK, NPM1 and ANXA2. Of these, HSPA8, HSPA9, PKM, HNRNPA1, NPM1, ANXA2 are already reported to be associated with LN metastasis in GBC or other cancers (Fig. 5).

### Clinical verification using Western blot and IHC analysis

**Fig. 6:**
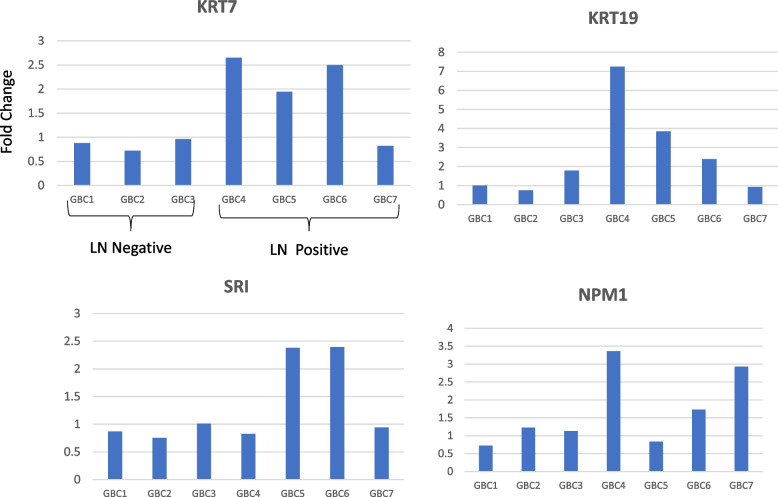
Altered levels of functionally relevant proteins in individual GBC patients as observed in quantitative proteomics data. The bar diagram showing the upregulated levels of KRT7, KRT19, SRI, NPM1 GBC LN positive GBC. LN Negative- GBC 1–3, LN Positive- GBC 4–7

Based on the significant fold change in LN positive GBC in mass spectrometric data and literature survey for their functional relevance and association with LN metastasis in cancer, we selected four proteins namely KRT7, KRT19, SRI and NPM1 for verification using Western blot analysis. The expression level of these proteins as per the quantitative proteomics data is shown in Fig. 6.

**Fig. 7:**
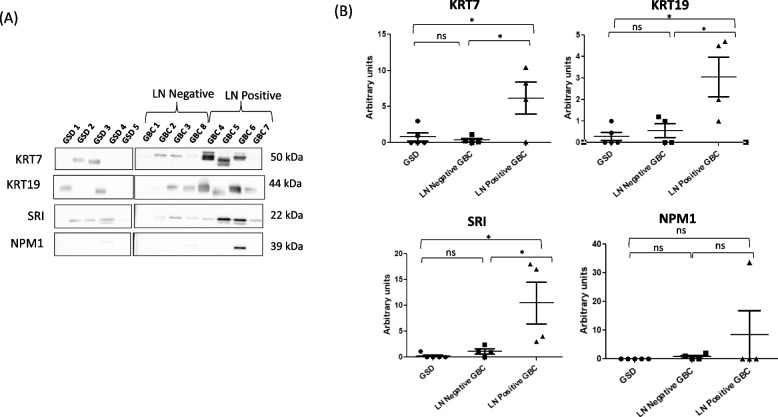
KRT7, KRT19, SRI and NPM1 protein expression in the LN positive GBC, LN negative GBC and GSD cases. (**A**) Western blot images showing expression of KRT7, KRT19, SRI and NPM1 in the individual tissue samples from three groups. (**B**) A significant overexpression (*p* value < 0.05) of KRT7, KRT19 and SRI was found in LN positive GBC cases in comparison to LN negative GBC cases or GSD cases (non-tumor control). No significant change in expression was observed for NPM1 in LN positive cases

We found a significant overexpression of three proteins, KRT7, KRT19 and SRI, with *p* value 0.03, 0.04 and 0.04 respectively while NPM1 did not show any significant difference in expression between the two groups. Western blot results are shown in Fig. 7 and the full-length blot images are presented in Supplementary Fig. S5.

**Fig. 8:**
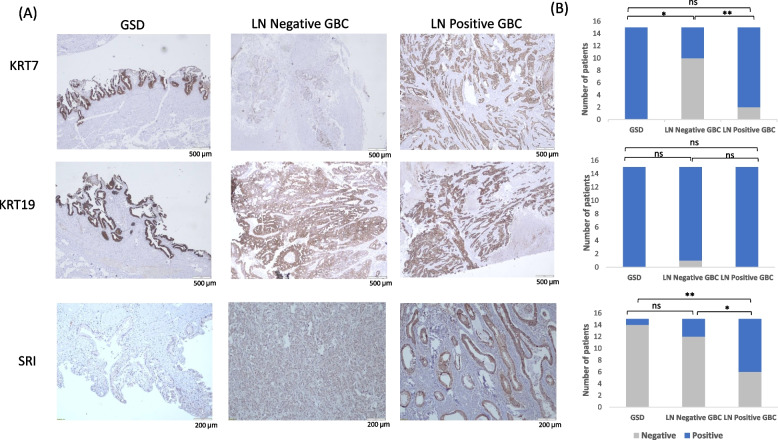
IHC analysis to study the expression of KRT7, KRT19 and SRI in the GBC cases and control group. (**A**) Representative IHC images showing the expression of KRT7, KRT19 and SRI in controls, LN Negative GBC and LN Positive GBC cases. IHC was performed on formalin-fixed paraffin-embedded (FFPE) individual tissue sections of 15 controls (GSD cases with no dysplasia), 15 LN Negative GBC cases and 15 LN Positive GBC cases. The IHC results showed that the expression of KRT7 was found to be ‘positive’ in 100% GSD cases, 33% LN negative GBC and 86% LN positive GBC. The expression of KRT19 was found to be ‘positive’ in 100% GSD cases, 93% LN negative GBC and 100% LN positive GBC. The expression of SRI was found to be ‘positive’ in 6% GSD cases, 20% LN negative GBC and 60% LN positive GBC. (**B**) The statistical analysis between LN positive vs LN negative GBC showed a significant difference in KRT7 and SRI expression (*p* value 0.002 and 0.02 respectively) while KRT19 did not show any significant difference in expression. The *p* values ≤ 0.05, ≤ 0.01 are marked with ‘*’ and ‘**’ respectively. The scale bar is shown as white line. IHC scoring is shown in Methodology section 2.8. GSD- Gallstone disease, LN- Lymph node

Based on the Western blot results, we performed IHC analysis to study the expression of three proteins, KRT7, KRT19 and SRI, in GSD (non-tumor control), LN negative GBC and LN positive GBC. The expression of KRT7 was found to be ‘positive’ in 100% GSD cases, 33% LN negative GBC and 86% LN positive GBC. KRT19 expression was found to be ‘positive’ in 100% GSD cases, 93% LN negative GBC and 100% LN positive GBC. The expression of SRI was found to be ‘positive’ in 6% GSD cases, 20% LN negative GBC and 60% LN positive GBC. Figure 8A shows the representative IHC images of controls, LN negative GBC and LN positive GBC. The statistical analysis between LN positive vs LN negative GBC showed a significant difference in KRT7 and SRI expression (*p* value 0.002 and 0.02 respectively) while KRT19 did not show any significant difference in expression in LN positive GBC (Fig. 8B).

## Discussion

LN metastasis is a major prognostic factor for patients with GBC as cancer cells from LNs may spread to other metastatic sites by lymphatic or blood vessels. It would be important to carry out an indepth analysis to understand the molecular processes associated with LN metastasis. Here, we have used iTRAQ-based quantitative proteomics approach and analyzed the differential proteome in LN metastatic GBC which led to the identification of 58 DEPs. Of these, 6 proteins are earlier reported to be associated with LN metastasis in GBC (KRT7, KRT19, VIM, PKM, LASP-1, TYMP), 33 proteins are associated with LN metastasis in other cancers (NPM1, SRI, FABP5, PPIA, HSPA9, AOC3, ANXA2, ANXA5, ANXA10, TAGLN2, HNRNPK, CAPG) while 19 proteins (DLST, MDH2, CKMT1A, APOC3, RDH16, VCAN) are novel to LN metastasis in cancer (Supplementary Table S4). Out of 58 proteins, five proteins (KRT7, KRT17, SRI, NPM1 and HNRNPA2B1) showed overexpression (≥ two-fold, *p* value < 0.05) in ≥ 50% of the LN positive patients and ≤ 1.3 fold change in LN negative patients. Three of these proteins (KRT7, KRT17 and SRI) are cytoskeleton proteins or cytoskeletal associated proteins and two proteins (NPM1 and HNRNPA2B1) are nuclear proteins. We further analyzed the expression of four proteins, KRT7, KRT19, SRI and NPM1 by Western blot and/or analysis.

The various components of ‘cytoskeleton and their associated proteins’ are well integrated in normal cells, however, remodeling of these proteins is well-established in cancer cells. Cytoskeleton proteins not only facilitate the invasion and migration of tumor cells but also regulate their intercellular signaling to facilitate tumor progression [25]. Some of the cytoskeleton proteins such as actin, vimentin, cytokeratins, transgelins are already reported to be involved in LN metastasis in various cancers [26-29]. In the present study, we observed cytoskeleton and associated proteins (KRT7, KRT17, KRT19, KRT6A, SRI) to be significantly overexpressed in LN positive GBC (Fig. 4A).

Cytokeratins (CKs or KRTs), the major group of proteins identified in this study, belongs to the intermediate filament (IF) protein family and epithelial cell markers that are routinely used in cancer diagnostics. It has been reported that cytokeratins release from proliferating or apoptotic cells, distinctly reflecting ongoing cell activity which makes them useful markers for epithelial malignancies [30]. Increased tissue expression levels of KRT7 is reported to be associated with LN metastasis in gallbladder cancer, lung cancer and colorectal cancer patients [28, 31, 32], KRT17 in gastric cancer, esophageal carcinoma and papillary thyroid carcinoma [33-35], KRT6A in lung adenocarcinoma [36], KRT19 in adenocarcinoma of gallbladder [37], thoracic tumors [38]. KRT7 and KRT19 staining of lymph nodes is reported to have a potential in detecting micrometastatic foci in regional lymph nodes of patients with GBC [12, 32] and cervical cancer [39] respectively. KRT19 expression was found to be the independent prognostic factor for hepatocellular carcinoma [40] and pancreatic ductal adenocarcinoma with LN metastasis [10]. Wang et al. reported KRT17 knockdown inhibited the invasion and proliferation of lung cancer cells. Moreover, its overexpression upregulated the activity of β-catenin and expression levels of Wnt target genes, such as cyclin D1, c-Myc, and MMP7 and also promoted EMT by increasing the levels of Vimentin [41]. Liu et al., 2019 explored the potential of KRT17 in proliferation and metastasis in esophageal squamous carcinoma. The volume and weight of KRT17 knockout tumors were smaller than the controls in vivo [35]. Here, we analyzed the expression of KRT7 and KRT19 by Western blot and IHC analysis. We found a significant overexpression of both the proteins in LN positive GBC in comparison to LN negative or GSD cases by Western blot analysis. However, IHC analysis confirmed the overexpression of KRT7 in LN positive GBC in comparison to LN negative GBC while KRT19 did not show any difference between the two groups. The previous studies on CK7 expression have been carried out in lymph node tissue (metastatic vs non-metastatic lymph nodes). Here, we confirm its overexpression in the primary tumor from LN positive GBC cases. All the GSD cases showed high expression of KRT7 and KRT19. The ‘low’ expression of KRT7 and KRT19 in GSD cases observed in proteomics data and Western blot data could be due to the presence of mixed tissue (epithelial mucosa, muscle tissue, connective tissue).

We also found sorcin, a cytoskeleton-associated protein, to be differentially expressed in LN positive GBC in the proteomics dataset. Sorcin is a 22 kDa soluble resistance related calcium-dependent protein reported to be involved in multidrug resistance in cancer [42]. This protein is associated with vimentin, a cytoskeletal protein already reported to be linked to LN metastasis in GBC. Deng et al.reported the association of sorcin with LN metastasis in gastric cancer patients by immunohistochemistry analysis [43]. It is well reported to play a role in EMT via downregulating the levels of epithelial marker ‘E-cadherin’ and upregulating the levels of mesenchymal marker ‘vimentin’ [44]. Sorcin exert its oncogenic effect by regulating key molecules such as VEGFs, MMPs, NF-κB, ERK1/ 2, CTSZ, Akt, STAT3, caspases involved in carcinogenesis and invasion of tumor cells and modulating signaling pathways including ERK, MAPK/ERK, and PI3K/Akt in various cancers. Supporting this fact, Hu et al.reported that sorcin silencing led to acquisition of epithelial-like morphology, attenuation of EMT and suppression of breast cancer metastasis in vivo [33]. Tong et al.reported the regulatory role of sorcin in EMT in colorectal cancer [45]. In the present study, the overexpression of SRI in LN positive GBC was confirmed by Western blot and IHC analysis. Sorcin is reported to be localized in nucleus and cytoplasm in various cancers as per HPA data (https://www.proteinatlas.org/ENSG00000075142-SRI/pathology). We observed both nuclear and cytoplasmic expression of sorcin in GBC. We found a significant difference in nuclear SRI expression in LN positive GBC in comparison to LN negative GBC, however, no significant difference in SRI expression was observed among these two groups when both nuclear and cytoplamic expression were considered. Deng et al.reported its cytoplasmic expression in gastric cancer and was associated with LN metastasis [43], whereas, we find its nuclear expression to be linked to LN metastasis in GBC. The role of nuclear SRI in LN metastasis is not clear and needs to be explored further.

We also observed an overexpression of two nuclear proteins, HNRNPA2B1 and NPM1 in our quantitative proteomics data. HNRNPA2B1 is an RNA binding protein involved in the transcription, splicing processing, transport, stability, telomere maintenance and DNA repair and its overexpression is reported to mediate EMT in different cancers [46, 47]. Higher HNRNPA2B1 gene expression was found to be associated with LN metastasis in esophageal carcinoma [48]. NPM1 is a nucleocytoplasmic shuttling protein having an essential role in cellular processes such as maintenance of genomic stability, DNA repair, centrosome duplication, ribosome biogenesis, cell cycle progression and regulation of activity of tumor suppressor genes p53 and ARF [49]. Correlation of higher NPM1 expression with LN metastasis was also found in patients with oral squamous carcinoma and colon cancer [50, 51]. Liu et al.reported an increased expression of NPM1 and its association with LN metastasis poor survival of colon cancer patients. Small interfering RNA (siRNA) of NPM1 inhibited migration and invasiveness of metastatic colon cancer in the HCT116 cell line [51]. Zhang et al.also reported inhibition of proliferation of HepG2 cells on NSC348884 treatment (a small molecular inhibitor of NPM1) [52]. Qi et al.explored the potential of NSC348884 as an anticancer drug and reported upregulation of p53 and apoptosis in a dose-dependent manner after treatment of several different cancer cell types with the inhibitor. NPM1 inhibitor also has a synergistic effect with chemotherapeutic drugs [53]. The expression of NPM1 was analyzed using Western blot analysis and we observed the overexpression of NPM1 in one of the LN positive GBC in comparison to LN negative GBC (25% positivity), however, based on the statistical analysis there was no significant difference in expression between LN positive GBC vs LN negative GBC.

Over all, the study identified a total of 58 proteins associated with LN metastasis in GBC which includes the cytoskeleton proteins and nuclear proteins. The overexpression of KRT7 and SRI in LN positive GBC in comparison to LN negative GBC was confirmed by Western blot and IHC analysis. The limitations include the low sample size used in the study. Also, the verification was restricted to the primary tumor and was not performed in the metastatic lymph nodes. KRT7 has been previously reported for its potential use in detection of micrometastasis in GBC and our study confirmed high positivity rate in primary tumor (gallbladder) from LN metastatic GBC. Further, the functional role of SRI using knockdown studies may be investigated in GBC cell lines for their potential as therapeutic targets for LN positive GBC cases.

## Supplementary Information

**Additional file 1: Supplementary Table S1.** Clinical parameters of patients such as age, gender, TNM, stage, grade, white cell count, liver enzymes (AST/ ALT/ ALP), bilirubin and co-morbidities (jaundice, pulmonary tuberculosis, asthma, diabetes melitus, hypertension (HTN), loss of appetite(LOA) and LOW- Loss of weight; Foot note: NA- Not applicable, (-) Not available. **Supplementary Table S2.** List of 58 DEPs in LN Positive GBC. The proteins with ≥2.0 fold change and adjusted *p* value <0.05 were used as DEPs. The details are provided in the Methods section. The Table describes the total number of proteins and peptides identified in mass spectrometry runs along with their quantity values. **Supplementary Table S3.** Bioinformatic analysis of 58 DEPs in LN positive GBC using STRING databse. (A) Top 10 Molecular Functions (B) Top 10 reactome pathways. **Supplementary Table S4.** Literature survey of 58 DEPs for their association with LN metastasis in cancer. A total of 6 proteins are reported in GBC, 33 proteins in other cancers and 19 were found to be novel to cancer with respect to LN metastasis.

**Additional file 2: Supplementary Figure S1.** Experimental design of iTRAQ-based quantitative proteomic analysis for identification of differentially expressed proteins in LN Metastatic GBC. **Supplementary Figure S2.** SDS-PAGE profile of tissue lysate GBC Stage IIIA and IIIB and control (GSD). A total of 15 µg protein was resolved on 12% gel and stained with Coomassie Brilliant Blue R250 to visualize the protein bands. The protein load for different samples was normalized based on the total density of proteins in each lane. **Supplementary Figure S3.** Venn diagram showing proteins identified in four technical replicate runs. We found a total of ~700 proteins in each replicate, of which 468 proteins were identified by all four technical replicates. Venn diagram was prepared using the BioVenn software. **Supplementary Figure S4.** Volcano plot showing DEPS in individual GBC patients. The volcano map was prepared by using log2 fold change and -log10 (p-value) as the co-ordinates and significant fold change ≥ 2.0 and p-value <0.05 were considered to screen the proteins. Dots in orange, blue and grey represents proteins that are upregulated, downregulated and unchanged respectively. GBC- Gallbladder cancer. **Supplementary Figure S5.** Full-length blot images of Fig. 7 for the expression of KRT7, KRT19, SRI and NPM1 in the individual tissue samples from LN positive GBC, LN negative GBC and GSD cases. For NPM1, the main image includes a) the lane with MW marker from the ‘low exposure’ image and b) other lanes showing NPM1 expression from the blot with ‘high exposure’. The pooled tissue lysate from GSD or LN negative or LN positive GBC was used for Negative control (the blot without primary antibody). Negative control data is not presented in the main image. The cropping of the blot images is indicated with red dashed line.

## Data Availability

All data generated or analyzed for this study is included in the main article and supplementary information files and is also available with the corresponding author.
