# Supplementary material for: Tissue proteome analysis for profiling proteins associated with lymph node metastasis in gallbladder cancer
Source: BMC Cancer. 2023 May 4;23:402. doi: 10.1186/s12885-023-10840-3 (PMC10161508; doi:10.1186/s12885-023-10840-3)
Supplement: Supplementary file 2 — Additional file 2: Supplementary Figure S1. Experimental design of iTRAQ-based quantitative proteomic analysis for identification of differentially expressed proteins in LN Metastatic GBC. Supplementary Figure S2. SDS-PAGE profile of tissue lysate GBC Stage IIIA and IIIB and control (GSD). A total of 15 µg protein was resolved on 12% gel and stained with Coomassie Brilliant Blue R250 to visualize the protein bands. The protein load for different samples was normalized based on the total density of proteins in each lane. Supplementary Figure S3. Venn diagram showing proteins identified in four technical replicate runs. We found a total of ~700 proteins in each replicate, of which 468 proteins were identified by all four technical replicates. Venn diagram was prepared using the BioVenn software. Supplementary Figure S4. Volcano plot showing DEPS in individual GBC patients. The volcano map was prepared by using log2 fold change and -log10 (p-value) as the co-ordinates and significant fold change ≥ 2.0 and p-value <0.05 were considered to screen the proteins. Dots in orange, blue and grey represents proteins that are upregulated, downregulated and unchanged respectively. GBC- Gallbladder cancer. Supplementary Figure S5. Full-length blot images of Fig. 7 for the expression of KRT7, KRT19, SRI and NPM1 in the individual tissue samples from LN positive GBC, LN negative GBC and GSD cases. For NPM1, the main image includes a) the lane with MW marker from the ‘low exposure’ image and b) other lanes showing NPM1 expression from the blot with ‘high exposure’. The pooled tissue lysate from GSD or LN negative or LN positive GBC was used for Negative control (the blot without primary antibody). Negative control data is not presented in the main image. The cropping of the blot images is indicated with red dashed line. [file 12885_2023_10840_MOESM2_ESM.pptx]

## Slide 1
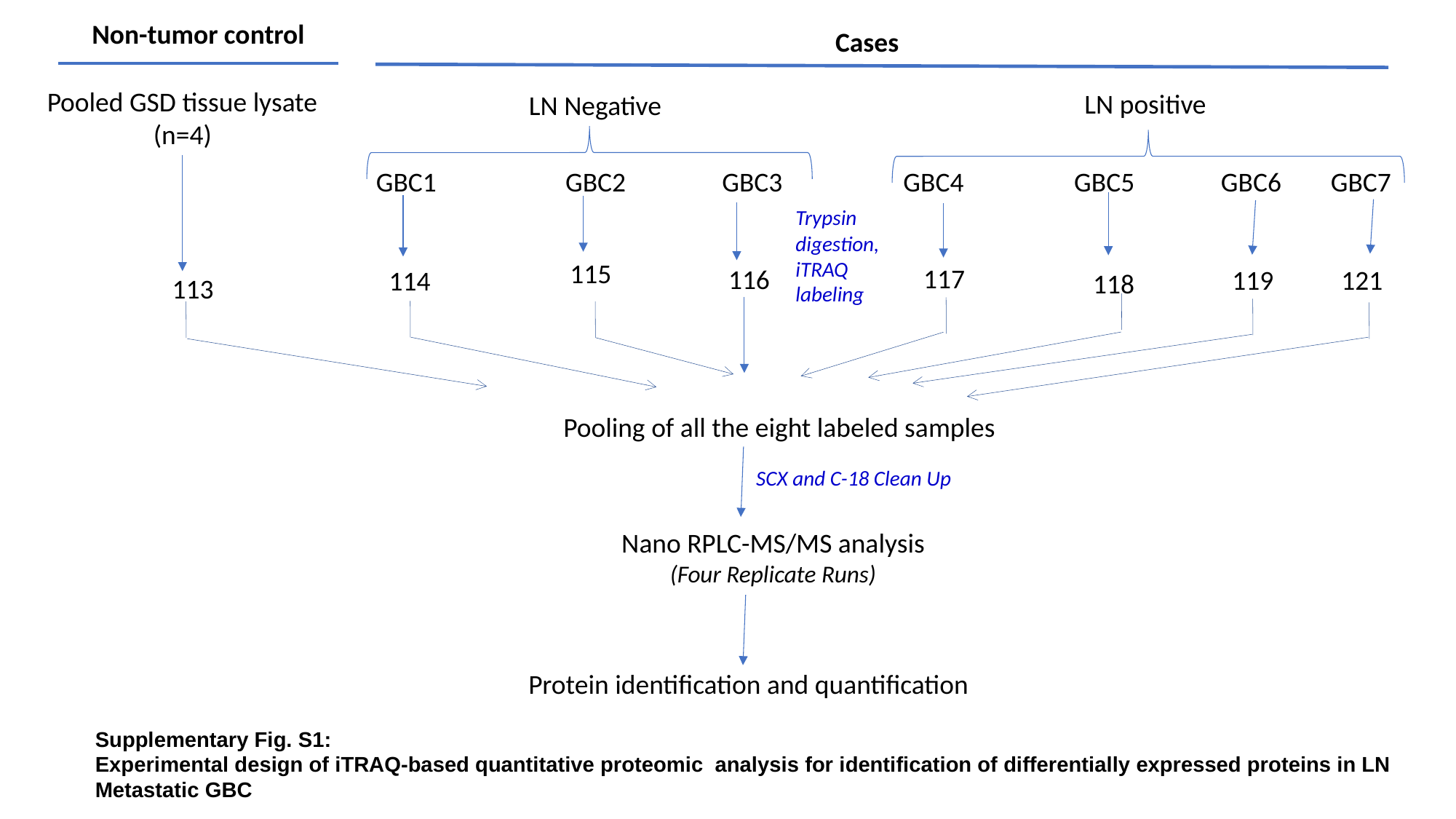

Non-tumor control
Cases
Pooled GSD tissue lysate
(n=4)
LN positive
LN Negative
 GBC1 	 GBC2	 GBC3	 	GBC4	 GBC5 GBC6 GBC7
Trypsin digestion, iTRAQ labeling
115
117
116
119	121
114
118
113
Pooling of all the eight labeled samples
SCX and C-18 Clean Up
Nano RPLC-MS/MS analysis
(Four Replicate Runs)
Protein identification and quantification
Supplementary Fig. S1:
Experimental design of iTRAQ-based quantitative proteomic analysis for identification of differentially expressed proteins in LN Metastatic GBC

## Slide 2
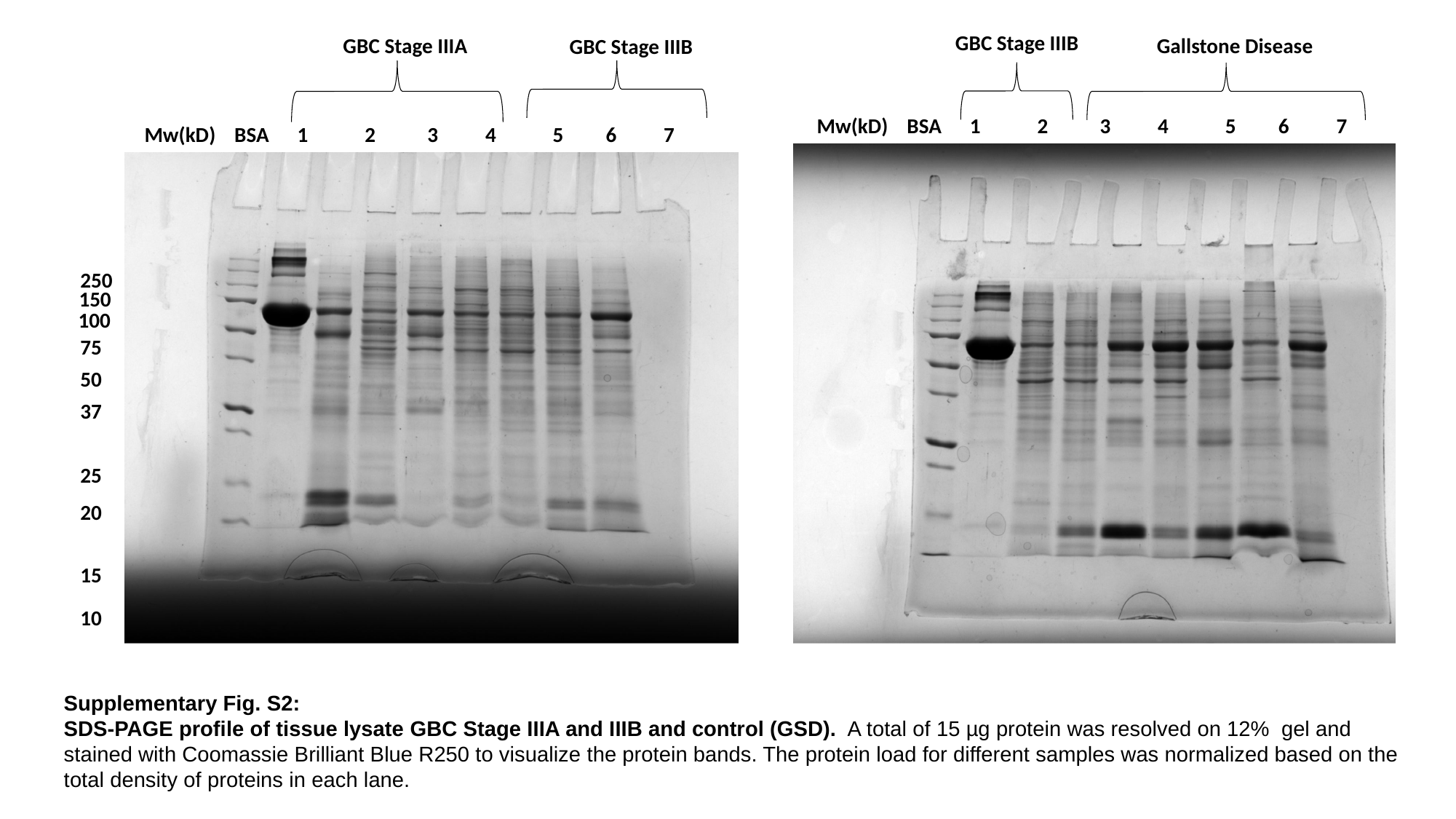

GBC Stage IIIB
Gallstone Disease
GBC Stage IIIA
GBC Stage IIIB
Mw(kD) BSA 1 2 3 4 5 6 7
Mw(kD) BSA 1 2 3 4 5 6 7
250
150
100
75
50
37
25
20
15
10
Supplementary Fig. S2:
SDS-PAGE profile of tissue lysate GBC Stage IIIA and IIIB and control (GSD). A total of 15 µg protein was resolved on 12% gel and stained with Coomassie Brilliant Blue R250 to visualize the protein bands. The protein load for different samples was normalized based on the total density of proteins in each lane.

## Slide 3
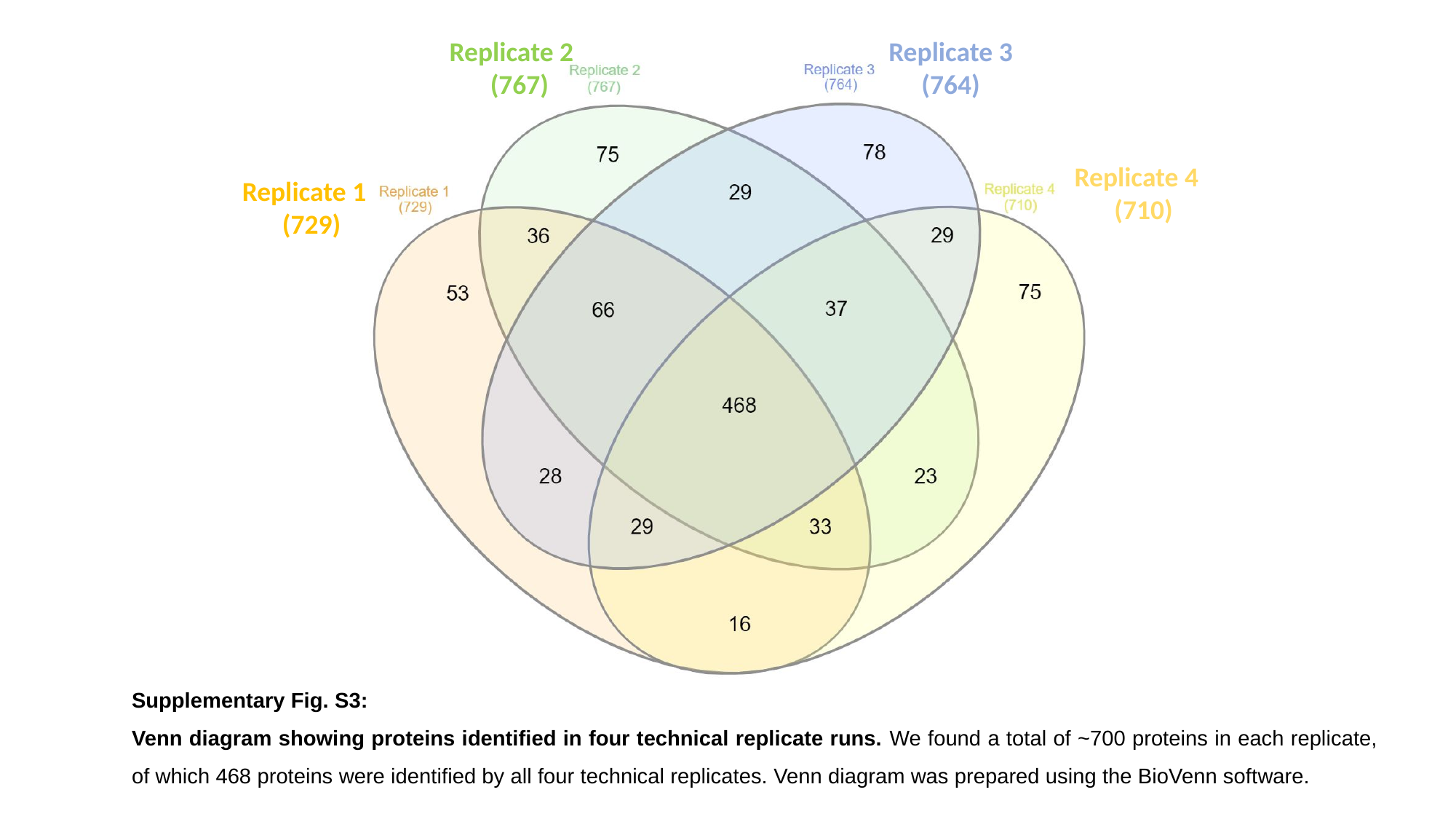

Replicate 2
(767)
Replicate 3
(764)
Replicate 4
(710)
Replicate 1
(729)
Supplementary Fig. S3:
Venn diagram showing proteins identified in four technical replicate runs. We found a total of ~700 proteins in each replicate, of which 468 proteins were identified by all four technical replicates. Venn diagram was prepared using the BioVenn software.

## Slide 4
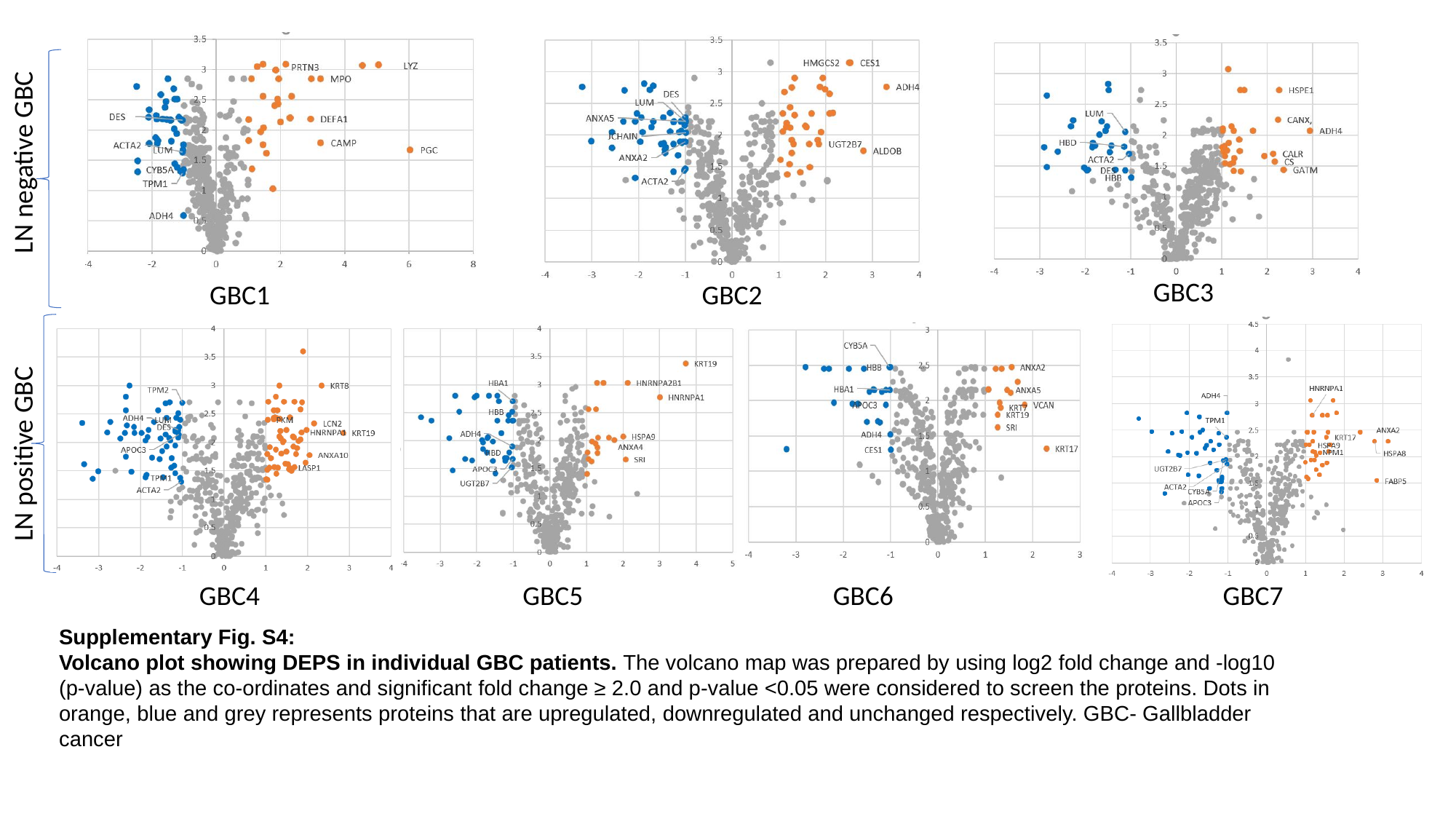

LN negative GBC
GBC3
GBC1
GBC2
LN positive GBC
GBC5
GBC6
GBC4
GBC7
Supplementary Fig. S4:
Volcano plot showing DEPS in individual GBC patients. The volcano map was prepared by using log2 fold change and -log10 (p-value) as the co-ordinates and significant fold change ≥ 2.0 and p-value <0.05 were considered to screen the proteins. Dots in orange, blue and grey represents proteins that are upregulated, downregulated and unchanged respectively. GBC- Gallbladder cancer

## Slide 5
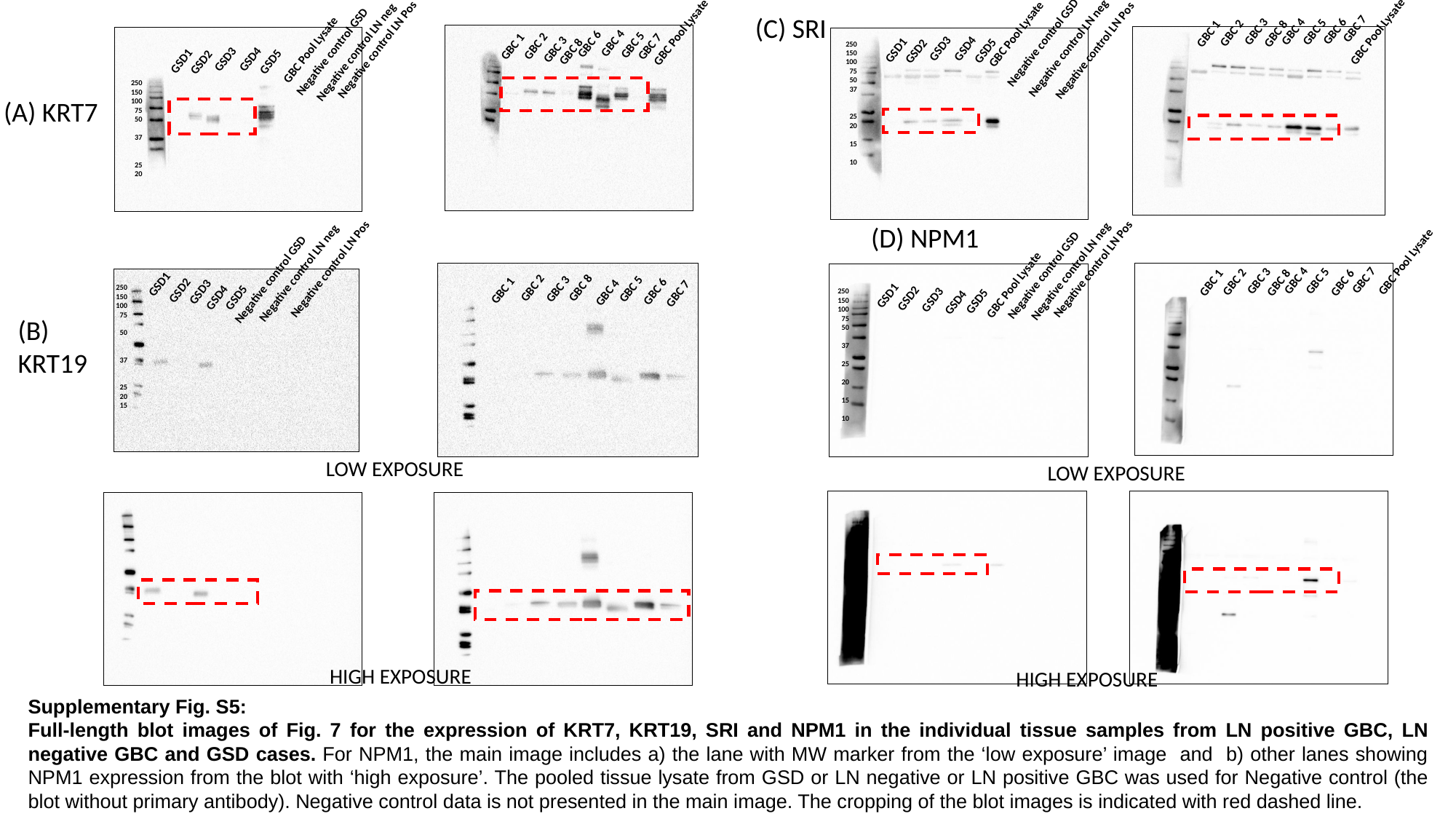

Negative control GSD
GBC Pool Lysate
GBC Pool Lysate
GBC Pool Lysate
GBC 7
Negative control GSD
(C) SRI
GBC 6
GBC 4
Negative control LN neg
GBC 3
GBC 5
Negative control LN Pos
GBC 2
GBC 8
GBC 1
Negative control LN Pos
Negative control LN neg
GBC Pool Lysate
GBC 4
GBC 6
GBC 5
GBC 2
GBC 7
GBC 1
GBC 3
GBC 8
GSD3
GSD4
GSD1
GSD2
GSD5
250
150
100
75
50
37
25
20
15
10
GSD3
GSD4
GSD1
GSD2
GSD5
250
150
100
75
50
37
25
20
(A) KRT7
(D) NPM1
Negative control LN Pos
Negative control LN Pos
Negative control GSD
GBC Pool Lysate
Negative control LN neg
Negative control LN neg
Negative control GSD
GBC Pool Lysate
GBC 7
GBC 4
GBC 3
GBC 5
GBC 2
GBC 8
GBC 6
GBC 1
GBC 2
GBC 8
GBC 3
GBC 5
GSD1
GBC 1
GBC 6
GBC 4
GBC 7
GSD2
GSD3
250
150
100
75
50
37
25
20
15
GSD1
GSD2
GSD4
250
150
100
75
50
37
25
20
15
10
GSD3
GSD5
GSD4
GSD5
(B) KRT19
LOW EXPOSURE
LOW EXPOSURE
HIGH EXPOSURE
HIGH EXPOSURE
Supplementary Fig. S5:
Full-length blot images of Fig. 7 for the expression of KRT7, KRT19, SRI and NPM1 in the individual tissue samples from LN positive GBC, LN negative GBC and GSD cases. For NPM1, the main image includes a) the lane with MW marker from the ‘low exposure’ image and b) other lanes showing NPM1 expression from the blot with ‘high exposure’. The pooled tissue lysate from GSD or LN negative or LN positive GBC was used for Negative control (the blot without primary antibody). Negative control data is not presented in the main image. The cropping of the blot images is indicated with red dashed line.
